# Ritual Risk: Incense Use and Cardiovascular Mortality

**DOI:** 10.1289/ehp.122-A336

**Published:** 2014-12-01

**Authors:** Nancy Averett

**Affiliations:** Nancy Averett writes about science and the environment from Cincinnati, OH. Her work has been published in *Pacific Standard*, *Audubon*, *Discover*, *E/The Environmental Magazine*, and a variety of other publications.

Numerous studies have examined exposures to indoor combustion products such as secondhand smoke and emissions from burning of solid fuels. However, only a few have examined incense burning as a potential health threat, even though incense is commonly used for religious and ritual purposes in China, Taiwan, Singapore, India, and Middle Eastern nations.^1^^,^^2^ In this issue of *EHP*, investigators report an association between long-term incense use and increased cardiovascular mortality.^1^

**Figure:**
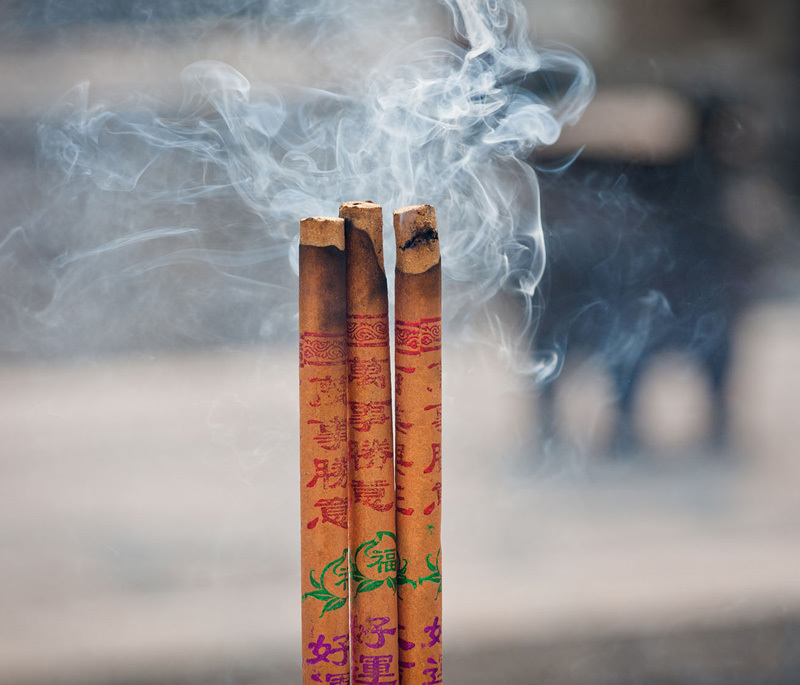
Although the ritual use of incense is common around the world, few studies have investigated its impact on indoor air quality and human health. © Fotokon/Shutterstock

The study used data from the Singapore Chinese Health Study, which enrolled a cohort of 63,257 Chinese adults aged 45–74 years between 1993 and 1998. The authors identified cardiovascular deaths of cohort members via a nationwide death registry, checking the registry yearly through 31 December 2011. They stratified their analysis for factors such as smoking history, education level, baseline history of cardiovascular disease, and gender. They also performed a sensitivity analysis to examine potential confounding by exposure to secondhand smoke.

More than three-quarters of the participants reported currently using incense, and another 13% were former users. Most had used incense daily for at least 20 years, typically keeping it burning intermittently throughout the day. The authors estimated that current long-term incense users had a 12% increased risk of cardiovascular mortality compared with former and never users, including a 19% increased risk for stroke and a 10% increased risk for coronary heart disease.^1^

Previous studies reported concentrations of volatile organic compounds and particulate matter in incense emissions similar to those in cigarette smoke.^3^^,^^4^ Others showed that long-term exposure to incense smoke increased blood vessel inflammation and affected blood flow in rats.^5^ *In vitro* studies have indicated adverse impact to human coronary^6^ and lung cells.^4^ But this is the first study to provide epidemiological evidence of effects at the population level resulting from habitual day-to-day burning of incense at home, says senior author Woon-Puay Koh, an epidemiologist at Duke–National University of Singapore Graduate Medical School.

“This study is of particular significance given that cardiovascular disease is one of the most common chronic diseases in the population worldwide,” says Karin Yeatts, an epidemiologist at the University of North Carolina at Chapel Hill, who has studied indoor air quality in the Middle East. In contrast with outdoor air pollution, incense exposure may be easier for an individual to avoid, but Yeatts says education will be needed to help people understand the risks of these exposures, similar to educational campaigns about cigarette smoking.

Limitations to the study include lack of information on the type of incense burned and the use of ventilation during incense burning. In addition, participants were asked about their incense exposure only once—during recruitment—and no data were available for nonfatal coronary heart disease or stroke. A strength of the study is the cohort study design, which provides evidence that the exposure of interest preceded the health outcome.

Koh published an earlier prospective study that found an association between incense use and upper respiratory cancer.^7^ Next she and her coauthors hope to look at cardiovascular risk biomarkers in relation to diseases such as diabetes mellitus and hypertension. It’s also unclear why the estimated impact on stroke was greater than that on heart disease, says first author An Pan, an epidemiologist at the National University of Singapore. “That could be very interesting to look at in terms of future research.”
